# Strategies to Investigate Ubiquitination in Huntington's Disease

**DOI:** 10.3389/fchem.2020.00485

**Published:** 2020-06-11

**Authors:** Karen A. Sap, Eric A. Reits

**Affiliations:** Department of Medical Biology, Amsterdam UMC, Amsterdam, Netherlands

**Keywords:** ubiquitin, Huntington's disease, huntingtin, neurodegenerative disease, proteasome, applications, toolbox

## Abstract

Many neurodegenerative disorders including Huntington's Disease are hallmarked by intracellular protein aggregates that are decorated by ubiquitin and different ubiquitin ligases and deubiquitinating enzymes. The protein aggregates observed in Huntington's Disease are caused by a polyglutamine expansion in the N-terminus of the huntingtin protein (Htt). Improving the degradation of mutant Htt via the Ubiquitin Proteasome System prior to aggregation would be a therapeutic strategy to delay or prevent the onset of Huntington's Disease for which there is currently no cure. Here we examine the current approaches used to study the ubiquitination of both soluble Htt as well as insolubilized Htt present in aggregates, and we describe what is known about involved (de)ubiquitinating enzymes. Furthermore, we discuss novel methodologies to study the dynamics of Htt ubiquitination in living cells using fluorescent ubiquitin probes, to identify and quantify Htt ubiquitination by mass spectrometry-based approaches, and various approaches to identify involved ubiquitinating enzymes.

## Introduction

### Protein Ubiquitination

Ubiquitin is a highly conserved small modular protein consisting of 76 amino acids that can be attached to other proteins as a post translational modification (PTM). Ubiquitination can modulate the properties of the target molecule, such as its cellular localization, interaction partners, protein activity, or it can send the target protein for degradation. As such ubiquitination controls major cellular processes including DNA repair, transcriptional regulation, cell cycle, protein turnover, and stress response. Protein ubiquitination is selectively mediated via the sequential action of three enzymes. First, ubiquitin is activated by a thioester bond formation with an internal active site cysteine residue of an E1 ubiquitin-activating enzyme in an ATP dependent manner. The activated ubiquitin is then transferred from the E1 enzyme to the cysteine residue of an E2 ubiquitin conjugating enzyme. Finally, an E3 ubiquitin ligase catalyzes the transfer of ubiquitin to specific target proteins (Hershko and Ciechanover, 1998). More than 600 different E3 ligases have been identified and they account for a high selectivity toward target protein ubiquitination. There are three main types of E3 ligases: RING/U-box ligases, RING-between-RING (RBR) ligases and HECT ligases, which have a different mode of action for the transfer of ubiquitin to target proteins. RING-type ligases transfer ubiquitin directly from the E2 enzyme to the target protein, while HECT-type ligases associate with ubiquitin via their active site cysteine before transferring it to the target protein. The RBR ligases use a combination of both strategies. These ligases harbor 2 RING domains, of which the RING1 domain is used to associate with an E2 enzyme while the RING2 domain associates with ubiquitin via its active site cysteine, before ubiquitin is transferred to the target protein.

Ubiquitin associates with its C-terminal glycine residue to target proteins. The canonical site of isopeptide bond formation for ubiquitin is the epsilon-amino group of a lysine residue. Other residues that can become a target for ubiquitination are protein N-terminal methionine residues, cysteine, serine, and threonine residues (McDowell and Philpott, 2013). Proteins can become ubiquitinated with a single ubiquitin molecule, also called monoubiquitination, or with two or multiple ubiquitin molecules each bound to different target sites, which is called multi-monoubiquitination. Alternatively, proteins can become polyubiquitinated when ubiquitin associates with other ubiquitin molecules on the target protein thereby forming a polyubiquitin chain. Several residues of ubiquitin can be used for polyubiquitin chain formation, including its N-terminal methionine residue (M1) as well as several internal lysine residues: K6, K11, K27, K29, K33, K48, and K63. These different chains display different structures, thereby giving different signals which determines the target protein's fate (Akutsu et al., 2016). For instance, K48 and K11 polyubiquitination are associated with proteasome-mediated degradation, while K6 and K63 polyubiquitination play a role in DNA repair. Polyubiquitin chains could be formed through one single ubiquitin linkage type (homotypic) or via different ubiquitin linkage types (heterotypic). Heterotypic polyubiquitin chains could furthermore be mixed and/or branched, and contain ubiquitin-like proteins, like Small Ubiquitin-like Modifier (SUMO). SUMO is a ubiquitin-like protein that also associates with lysine residues on target proteins, and thereby it can affect multiple cellular processes such as protein translocation, DNA damage response and cell cycle progression. SUMO-targeted ubiquitin ligases (STUBLs) can attach ubiquitin to proteins that are already SUMOylated and target them for ubiquitin-dependent degradation (Uzunova et al., 2007). Finally, ubiquitin can be modified with other PTMs such as phosphorylation, SUMOylation, acetylation, and neddylation (Swatek and Komander, 2016). This all together makes ubiquitination a versatile modification, also called the ubiquitin code, and is reviewed in great detail elsewhere (Yau and Rape, 2016).

Protein ubiquitination can be reversed by deubiquitinating enzymes or DUBs which can cleave the peptide or isopeptide bond between a conjugated ubiquitin molecule and the modified protein. In humans over 100 different DUBs have been identified and they show selectivity toward specific protein substrates or toward specific polyubiquitin linkage types. The latter category can be divided in different subgroups, including DUBs that cleave within chains (endo-DUB-activity), DUBs that remove ubiquitin molecules from the end of polyubiquitin chains (exo-DUB activity), DUBs that prefer a specific chain length, and finally, DUBs that cleave off entire polyubiquitin chains. In general, members of the USP family of DUBs show little specificity for polyubiquitin linkage types, while members of the OTU family often display preferences for diverse polyubiquitin chain types. For instance, OTUB1 has a preference for K48-polyubiquitin linkages, while OTUD7B/Cezanne cleaves K11 ubiquitin linkages. Polyubiquitin linkage type specific DUBs have been elegantly reviewed elsewhere (Clague et al., 2019), but it is clear that the action of DUBs can suppress the abundance of polyubiquitinated proteins in the cell, thereby influencing the regulation of important cellular processes such as protein degradation and thus protein turnover rates. The important role of (de)ubiquitinating enzymes in selective protein turnover is also reflected in numerous protein misfolding disorders when efficient ubiquitination and turnover of particular proteins is impaired either due to mutations in involved (de)ubiquitinating enzymes or in disease-related target proteins. Mutations in E3 ligases and DUBs are linked to particular disorders, including the DUB ataxin-3 (Spinocerebellar ataxia 3) (Kawaguchi et al., 1994) and the E3 ligase Parkin (familiar form of Parkinson's Disease) (Kitada et al., 1998), which affects efficient target protein recognition and subsequent ubiquitination, thereby limiting protein degradation via the proteasome and autophagy. In addition, mutations in disease-related proteins including the Htt protein in Huntington's Disease (HD) results in the generation of aggregation-prone protein (fragments). Many neurodegenerative disorders are hallmarked by intracellular protein aggregates, and while these aggregates or inclusion bodies (IBs) are being decorated with ubiquitin, the turn-over of the disease-related proteins is apparently not efficient enough to prevent their accumulation. Due to the monogenetic cause, the availability of numerous *in vitro* and *in vivo* models, and the various techniques to monitor intracellular protein aggregates, HD became a commonly-used model to study ubiquitination dynamics and the role of the Ubiquitin Proteasome System (UPS) in protein misfolding diseases.

### Huntington's Disease

HD is one of nine polyglutamine (polyQ) diseases, and is caused by an expansion of a CAG trinucleotide repeat in the exon-1 region of the *Huntingtin* (*Htt*) gene (MacDonald et al., 1993). The wild-type protein contains 6-35 polyQ repeats, while an expansion of more than 39 polyQ repeats in the mHtt protein causes HD (Finkbeiner, 2011). The polyQ expansion makes the protein aggregation prone and the aggregation of mHtt into IBs are a hallmark for the disease (Imarisio et al., 2008; Finkbeiner, 2011). The polyQ region is located close to the N-terminus of the Htt protein, which contains 17 N-terminal amino acids including 3 lysine residues, followed by the polyQ region and a polyproline region (Finkbeiner, 2011). Proteolysis of mHtt results in the formation of different mHtt protein fragments of which the N-terminal exon1 fragment containing the polyQ expansion was found to be the most pathogenic and is also observed in fibrillar aggregates in brains of HD patients (DiFiglia et al., 1997; Schilling et al., 2007; Landles et al., 2010) and overexpression of mutant Htt exon1 results in HD-like symptoms in mice (Mangiarini et al., 1996). The aggregation-prone fragments of mHtt can be present in cells as monomers, soluble oligomers or in insoluble aggregates including the insoluble IBs. The current model is that especially soluble oligomeric mHtt species are toxic to the cell, while large aggregates of mHtt sequester proteins from their normal cellular environment, thereby interfering with important processes such as transcriptional regulation (Schaffar et al., 2004), proteostasis (Park et al., 2013), and transport (Trushina et al., 2004). However, mHtt aggregates have also been described as protective as they reduce the level of toxic soluble mHtt species in the cell (Arrasate et al., 2004). Additionally, soluble mHtt induced apoptosis was found to be reduced by mHtt IB formation and led to a slower death by necrosis, which also suggests that IB formation functions as a rescue mechanism (Ramdzan et al., 2017).

**Figure 1:**
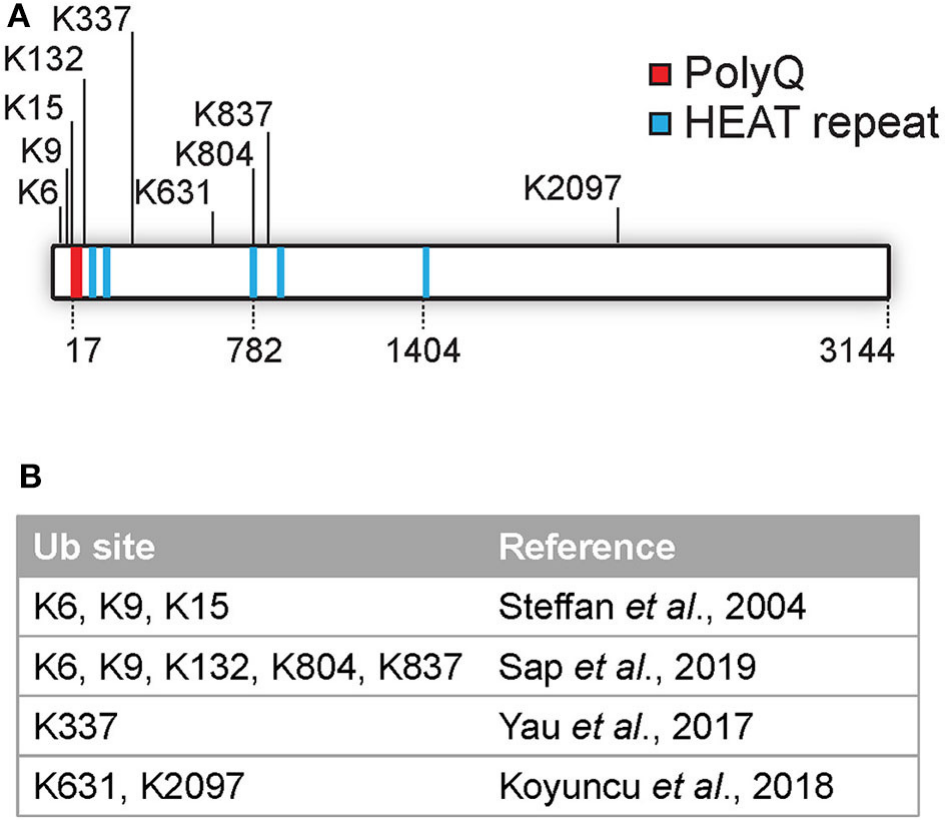
Overview of identified Htt ubiquitination sites**. (A)** Overview of identified Htt ubiquitination sites. **(B)** Overview of published studies on Htt ubiquitination site identification.

Several PTMs of Htt, including phosphorylation, SUMOylation, ubiquitination, acetylation, and palmitoylation have been identified (as reviewed by Ehrnhoefer et al., 2011; Saudou and Humbert, 2016). Many PTMs are localized at the N-terminal region of Htt, and include acetylation, SUMOylation, and ubiquitination at lysines 6, 9, and 15 and phosphorylation at threonine 3 as well as at serines 13 and 16. These modifications can affect the subcellular localization, aggregation and clearance of Htt (Steffan et al., 2004; Thompson et al., 2009; Maiuri et al., 2013; DeGuire et al., 2018). Most PTMs were found to be localized in clusters within predicted unstructured domains and not in the structured HEAT repeats as determined by using label free quantitative mass spectrometry (Ratovitski et al., 2017). In addition, mutations in various phosphorylation sites located in protease-sensitive domains on the Htt protein affected cellular toxicity, and are thus important functional regions (Arbez et al., 2017). While various studies have mapped numerous phosphorylation and acetylation sites, the number of identified ubiquitination sites in Htt is so far limited to K6, K9, K15, K132, K337, K631, K804, K837, and K2097 (Steffan et al., 2004; Yau et al., 2017; Koyuncu et al., 2018; Sap et al., 2019; Figures 1A,B). Ubiquitination and SUMOylation of the N-terminus of mHtt was found to affect both aggregation and HD pathology in cells although the mechanism is still unclear (Steffan et al., 2004).

### Htt Is a Target for Ubiquitin-Proteasome-System and Autophagy Dependent Degradation

Htt IBs are enriched with components of the protein quality control machinery including ubiquitin, proteasome complexes, and chaperones (DiFiglia et al., 1997; Wyttenbach et al., 2000). A decrease in proteostasis and quality control during aging are thought to play a role in the development and progression of HD, which may explain that the onset of HD starts typically at the 4th decade of life, although the mutant Htt protein is already expressed prior to birth (Arrasate and Finkbeiner, 2012). Several studies have shown that both wild-type Htt and mHtt can be degraded by both the UPS and via autophagosomal pathways. For example, mHtt protein fragments can be degraded by the proteasome, as shown by *in vitro* degradation assays using purified mHtt and proteasomes (Juenemann et al., 2013) or in cells (Bhat et al., 2014). Targeting mHtt to the proteasome by the N-end rule, or by using Atg5 knock-out MEF cells with impaired autophagy also shows efficient proteasomal degradation of mHtt (Juenemann et al., 2013). Inhibition of the proteasome increased the number of Htt aggregates in cells and in induced pluripotent stem cells (iPSCs) generated from HD-patients, which can be quantified by microscopy or filter retardation assays (Wyttenbach et al., 2000; Waelter et al., 2001; Koyuncu et al., 2018). Furthermore, protein levels of both normal and mutant full length Htt were increased by proteasome inhibition in heterozygous iPSCs (Koyuncu et al., 2018). Interestingly, longer mHtt fragments (508 a.a.) appear to be better targets for proteasomal degradation when compared to smaller fragments including Htt exon1 (Bhat et al., 2014) which might be due to the presence of unstructured HEAT-like repeat motifs in mHtt that facilitate initiation of proteasomal degradation. Htt levels are also regulated via autophagy, as shown by autophagy inhibition by 3-methyladenine (3-MA) and bafilomycin which increased the level of soluble mHtt protein fragments in striatal cells, as observed by SDS-PAGE Western blot (WB). Also, the number of cells with aggregates increased upon these treatments, as observed by microscopy (Qin et al., 2003), although autophagy inhibition also impairs protein degradation via the UPS (Korolchuk et al., 2009). The p62/SQSTM1 protein plays a role in targeting polyubiquitinated protein aggregates for degradation via the autophagy pathway (Bjørkøy et al., 2005), but the accumulation of the p62/SQSTM1 protein due to autophagy inhibition can also inhibit the clearance of ubiquitinated proteins destined for proteasomal degradation. Stimulation of autophagy with rapamycin or serum reduction lowered the levels of the Htt protein, and fasting has been proposed in order to induce autophagy and thereby the clearance of mHtt (Ehrnhoefer et al., 2018). Concluding, Htt appears to be a target for both the UPS and autophagy, and enhanced selective degradation of mHtt via these pathways might be a therapeutic strategy to prevent or delay the onset of HD, with a key role for ubiquitin to target Htt for degradation. Here we give an overview of the current status of research focused on Htt ubiquitination, describe tools that are used to study ubiquitination of both soluble and insoluble mHtt, and discuss various developments including the development of novel tools such as proximity-dependent biotin identification (BioID), engineered ascorbate peroxidase (APEX), tandem ubiquitin binding entities (TUBEs), and proteolysis targeting chimeras (PROTACs).

## Tools to Study Ubiquitination of Soluble and Insoluble Htt

### Methods to Study Aggregated Htt Ubiquitination

The mHtt protein can be present in either a soluble or insoluble fraction, and there are several methods available that one can use to study the insoluble fraction. Mutant Htt aggregates and IBs can be visualized using microscopy assays including immunostaining and fluorescently-tagged proteins, and various biochemical methodologies can be used to study differences in levels of mHtt aggregation. When studying the ubiquitination of Htt it is of importance to separate the soluble and insoluble fractions efficiently as aggregated proteins might be ubiquitinated differently when compared to their soluble counterparts. The use of a mild lysis buffer, for instance buffers based on 1% Triton X-100, was found to be suitable to separate the soluble fraction from the insoluble fraction (Ochaba et al., 2018). In contrast, stronger detergents such as SDS can solubilize the outer layer of aggregates, which would subsequently contaminate the soluble fraction, although it is a suitable protocol to access and study the ubiquitination of the inner core of the aggregates (Juenemann et al., 2015). Two frequently used biochemical techniques to study Htt aggregation are the filter retardation assay or filter trap assay, and the soluble/insoluble assay or solubilization assay. With a filter retardation assay, lysates are filtered through a cellulose acetate membrane, whereby the soluble fraction goes through the filter, while the insoluble fraction, including the IBs, remains on the filter allowing subsequent immunostaining similar to WB analysis (Wanker et al., 1999). Determining ubiquitination of mHtt aggregates by fluorescence or electron microscopy and the filter retardation assay can be performed using antibodies directed to ubiquitin or directed to specific polyubiquitin linkages. However, the obtained signal could be derived from ubiquitinated Htt but also from co-sequestered proteins that are modified by ubiquitin, and is thus not a proof for direct ubiquitination of Htt itself. Here, the soluble/insoluble assay has the advantage over the filter retardation assay to study PTMs in both the soluble and insoluble fractions (Juenemann et al., 2015). Briefly, in this assay the cell lysates containing 1.5% SDS are boiled after which the soluble and insoluble fractions are separated by centrifugation, and subsequently the SDS-insoluble fraction is dissolved and solubilized in 100% formic acid (Carra et al., 2008). Another advantage of the soluble/insoluble assay is that the samples are resolved by SDS-PAGE which gives information about the molecular weight of the studied proteins. Each ubiquitin molecule adds ~8.5 kDa to the target protein, so an increased molecular weight of the protein of interest will be visible in the form of one or more higher molecular bands or a smear above the protein of interest when stained with an antibody against the protein of interest on a WB. Additional controls include the use of lysine-to-arginine (KR) mutants of the protein of interest, by which the potential ubiquitination sites of a protein of interest are mutated. The higher molecular bands should not appear if the protein of interest would have been ubiquitinated at the sites that were mutated. Lysine-to-arginine substitutions can also be used to identify ubiquitin sites for mHtt-exon1 (Steffan et al., 2004) which is described in more detail in paragraph Identification of Htt Ubiquitination Sites. Mutation of lysine residues to arginine residues can also be applied to the ubiquitin protein itself in order to study polyubiquitin linkage types of a protein of interest by expressing ubiquitin cDNA constructs with point mutations. For instance, a K48-only ubiquitin mutant, of which all lysines are mutated to arginines except for the lysine at position 48, can only make K48 homotypic polyubiquitin chains. In contrast, a K48R ubiquitin mutant, which contains all internal lysines except at the 48th amino acid where the lysine is replaced with an arginine, can make all polyubiquitin chains except for K48-linked chains. Such Ub mutants were used to study K48 and K63 polyubiquination in combination with stably expressed full length wtHtt (23Q) and mHtt (120Q) in HEK293 cells (Bhat et al., 2014). When full-length Htt was immunoprecipitated under native conditions and analyzed for polyubiquitination by SDS-PAGE WB, mHtt turned out to be mainly ubiquitinated via K63-polyubiquitin. However, it is important to note that immunoprecipitations under native conditions co-purify other ubiquitinated material, and a pull-down experiment under denaturation conditions (e.g., with the overexpression of His-tagged ubiquitin variants and a strong lysis buffer) is more suitable as to gain insights into ubiquitin linkage types onto soluble Htt. In addition, overexpression of ubiquitin with point mutations has its own shortcomings as overexpression of ubiquitin mutants will affect and hamper many processes in the cell. Concluding, typical approaches that could be used to study the ubiquitination of mHtt IBs include visualization by microscopy, and biochemical approaches such as the filter retardation assay and the soluble/insoluble assay, in combination with ubiquitin detection via specific antibodies and the use of arginine-to-lysine mutants.

### Enrichment Protocols for Small Pools of Ubiquitinated Htt

**Figure 2:**
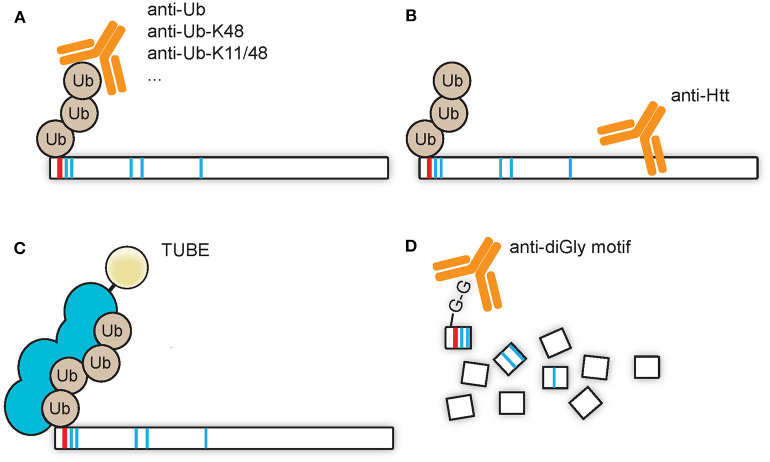
Enrichment strategies for ubiquitinated Htt. **(A)** Antibodies directed against ubiquitin or a polyubiquitin linkage type can be used to enrich ubiquitin-modified Htt from samples, after which the enriched Htt species can be visualized by SDS-PAGE WB with Htt-specific staining. This is the method of choice in order to detect specific ubiquitination of a substrate. **(B)** Antibodies directed against Htt can be used to enrich Htt and ubiquitin modified Htt species from samples, after which the enriched proteins can be visualized by SDS-PAGE WB with ubiquitin-specific staining. This method could give false positive results as ubiquitin-modified co-sequestered proteins could contribute to the obtained ubiquitin detection signal. **(C)** TUBEs, which display a preferential affinity for M1, K48, or K63 tetra-ubiquitin, could be used to enrich polyubiquitinated Htt from samples. **(D)** diGly-modified peptides, derived from ubiquitinated proteins after trypsin digestion, could be enriched from samples with antibodies directed against this diGly remnant motif, and subsequently detected by LC-MS/MS.

A low stoichiometry of protein ubiquitination can make it necessary to enrich the pool of ubiquitin-modified proteins from the total pool of proteins, for example with the use of pull downs via immunoprecipitation. A pull down directed against (poly)ubiquitin and subsequently visualized by SDS-PAGE WB with immunostaining for the protein of interest would be the best method to prove direct ubiquitination of the protein of interest (Figure 2A). Similarly, the overexpression of ubiquitin harboring an affinity tag could be used for pull downs under native conditions, such as with Myc, hemagglutinin (HA), FLAG, or glutathione S-transferase (GST) tags. Alternatively, pull downs under denaturing conditions, with for instance a poly-histidine (His) tag, could be done with the advantage of a reduction of the amount of co-purifying proteins. A pull down against Htt and a subsequent visualization of the (poly)ubiquitin signal might give a wrong impression due to putative co-purifying proteins which might be ubiquitinated (Figure 2B). Alternatively, TUBEs, which consists of a sequence of artificially generated and linked ubiquitin-associated (UBA) domains that recognize polyubiquitin linkages, can be used for pull downs (Figure 2C). The advantage of TUBEs over antibodies is that TUBEs protect the polyubiquitin chains from DUBs and proteasomal degradation in the cell lysate (Hjerpe et al., 2009). As described in the future perspectives paragraph Capturing Polyubiquitinated Proteins by Tandem Ubiquitin Binding Entities (TUBEs), TUBEs have to our knowledge not been applied to study Htt ubiquitination while it might yield valuable insight in the polyubiquitin landscape during HD, as the use of another isolated UBA domain, the UBA domain of ubiquilin-2, has been successfully applied to enrich K48-linked polyubiquitinated proteins from HD models and patient samples (Bennett et al., 2007). A different approach is the combination of immunoprecipitation and mass spectrometry in order to pull down so-called diGly peptides in order to define which lysines of proteins are ubiquitinated (Figure 2D). Ubiquitin associates with its C-terminal glycine residue to lysine residues of the target proteins, and upon digestion with trypsin a glycine-glycine (diGly) remnant motif derived from ubiquitin will remain on ubiquitin-modified lysine residues of proteins. The branched diGly motif is subsequently recognized by a specific antibody which could be used to enrich the diGly peptides from the samples and since the branched diGly motif on lysine residues results in mis cleavage of that specific lysine residue the site of ubiquitination can be identified using mass spectrometry (Kim et al., 2011; Wagner et al., 2011). The diGly peptides can be identified and quantified by LC-MS/MS, by which both ubiquitinated proteins and their ubiquitination sites can be characterized. The advantage of this technique is the unbiased nature and the direct proof of ubiquitination by mass spectrometry as well as a reduced complexity of the sample as compared to protein-targeted enrichments, as only the peptides that contain the diGly remnants are pulled down (Figure 2D). A disadvantage is the inability to discriminate between diGly remnants derived from ubiquitin and from ubiquitin-like proteins NEDD8 and ISG15. Recently an antibody named UbiSite has been generated which recognizes the 13 C-terminal amino acids of ubiquitin (Akimov et al., 2018). This antibody is used in Lys-C digested samples and could recognize ubiquitination on lysine residues as well as ubiquitination of protein N-termini. This antibody is thus specific for remnants derived from ubiquitin.

### Identification of Htt Ubiquitination Sites

#### Identification of Htt Ubiquitination Sites by Lysine-to-Arginine Mutants

Since lysine residues are the canonical sites for isopeptide bond formation with ubiquitin, mutation into residues that cannot become ubiquitinated, such as arginine residues, can reveal the lysine residues that function as sites for ubiquitin association. The lysine-to-arginine mutants are then compared to the original constructs to examine altered ubiquitination patterns. Htt exon1 contains 3 lysine residues, K6, K9, and K15, and several single and multiple lysine-to-arginine mutants have been generated in order to study SUMOylation and ubiquitination of Htt exon1 (Steffan et al., 2004). When these lysine-to-arginine mutants were used for pull downs in combination with overexpressed HIS-tagged SUMO and HIS-tagged ubiquitin, mutant Httex1 was shown to be modified both with SUMO-1 and ubiquitin, whereas mutation of all lysine residues did not show a signal for SUMOylation and ubiquitination. This indicates that Htt exon-1 was directly SUMOylated or ubiquitinated at either one or more of these lysine residues. Single and double mutations of the Httex1 lysine residues to arginines revealed that K6 and K9 were the main sites for ubiquitination and SUMOylation. Interestingly, mutation of the three lysines to arginines reduced HD pathogenicity, as expression of unmodified Httex1p 97QP resulted in a rough eye phenotype in *Drosophila*, while expression of the K6R, K9R, K15R mutant gave almost no detectable phenotype, and resulted in decreased abundance of the Htt exon1 protein which may indicate that ubiquitination is not a requirement for degradation.

#### Identification of Htt Ubiquitination Sites by Mass Spectrometry

Mass spectrometry has proven to be a powerful tool for the identification of PTMs, such as protein ubiquitination, and has been applied to characterize Htt ubiquitination sites. For instance, immunoprecipitation of the full-length Htt protein from HEK293 cells that overexpress Htt-Q100 followed by mass spectrometry revealed K631 and K2097 as Htt ubiquitination sites (Koyuncu et al., 2018). Another study identified K337 as ubiquitination site of endogenous Htt (Yau et al., 2017). As opposed to protein-level enrichment methods, the unbiased large-scale detection of ubiquitination sites in samples enriched for modified lysine-containing peptides has greatly enhanced the number of identified ubiquitination sites. These modified lysine-containing diGly peptides are pulled down with a K-ε-GG specific antibody, which is explained in more detail in paragraph Enrichment Protocols for Small Pools of Ubiquitinated Htt (Figure 2D) (Kim et al., 2011; Wagner et al., 2011). Pull down of diGly-modified peptides reduces the complexity of the sample as compared to a pull down with an antibody against the protein of interest or against ubiquitin. Furthermore, this technique can be combined with quantitative mass spectrometry approaches, and both qualitative and quantitative differences in ubiquitination can be found between different samples in an unbiased fashion. In this way K6, K9, K132, K804, and K837 were identified as ubiquitination sites of soluble full length Htt in brain lysates of 40 weeks old Q175 mice and wild-type controls (Sap et al., 2019). K6 and K9 were mainly ubiquitinated at the mutant soluble Htt protein, while K132, K804, and K837 were mainly ubiquitinated at the wild-type soluble Htt protein. This indicates that the polyQ expansion in the Htt protein affects ubiquitination.

## Studying the Dynamics of Htt Aggregate Ubiquitination

### Ubiquitin Is Dynamically Recruited to mHtt Aggregate IBs

Large intracellular Htt aggregates are often called IBs and are detected in the brain of HD-affected patients, especially in the cortex and striatum. These IBs were found to be decorated by ubiquitin as well as with other proteostasis-related proteins including proteasome complexes and chaperones, as shown in human postmortem cortical tissues, HD mouse model brain tissues and HD cell models (Davies et al., 1997; DiFiglia et al., 1997; Waelter et al., 2001). It is not clear yet which function ubiquitin fulfills at these IBs. Several microscopy-based studies revealed that the recruitment of ubiquitin is not essential for IB formation, since ubiquitination was not detected on nascent aggregates in mice brain tissue of juvenile R6/2 mice but only in later stages of disease, as demonstrated by using Ub antibody staining followed by microscopy (Davies et al., 1997; Gong et al., 2012). Furthermore, fluorescently-tagged ubiquitin (YFP-Ub) and Htt-exon1 (mCherry) were used to study the recruitment of ubiquitin to Htt exon1 IBs in living cells using fluorescent microscopy (Hipp et al., 2012). Upon transfection, Htt exon1 was initially diffusely distributed through the entire cytoplasm. However, when IBs became apparent, both IB size and fluorescence intensity increased rapidly, with the majority of fluorescently-labeled mHtt exon1 being recruited to IBs within 20–30 min. Interestingly, co-expressed YFP-tagged Ub was recruited toward mHtt exon1 IBs at a later stage when the IBs had already reached their mature size. This suggests that ubiquitination is not required for mHtt IB formation, and that Ub recruitment may depend on recruitment of ubiquitinating enzymes to IBs first. In addition, a 3xKR mutant Htt-exon1 with all lysines mutated to arginines and unable to form ubiquitin chains, can still form intracellular aggregates, indicating that mHtt IB formation is independent of ubiquitination of Htt itself (Juenemann et al., 2015).

**Figure 3:**
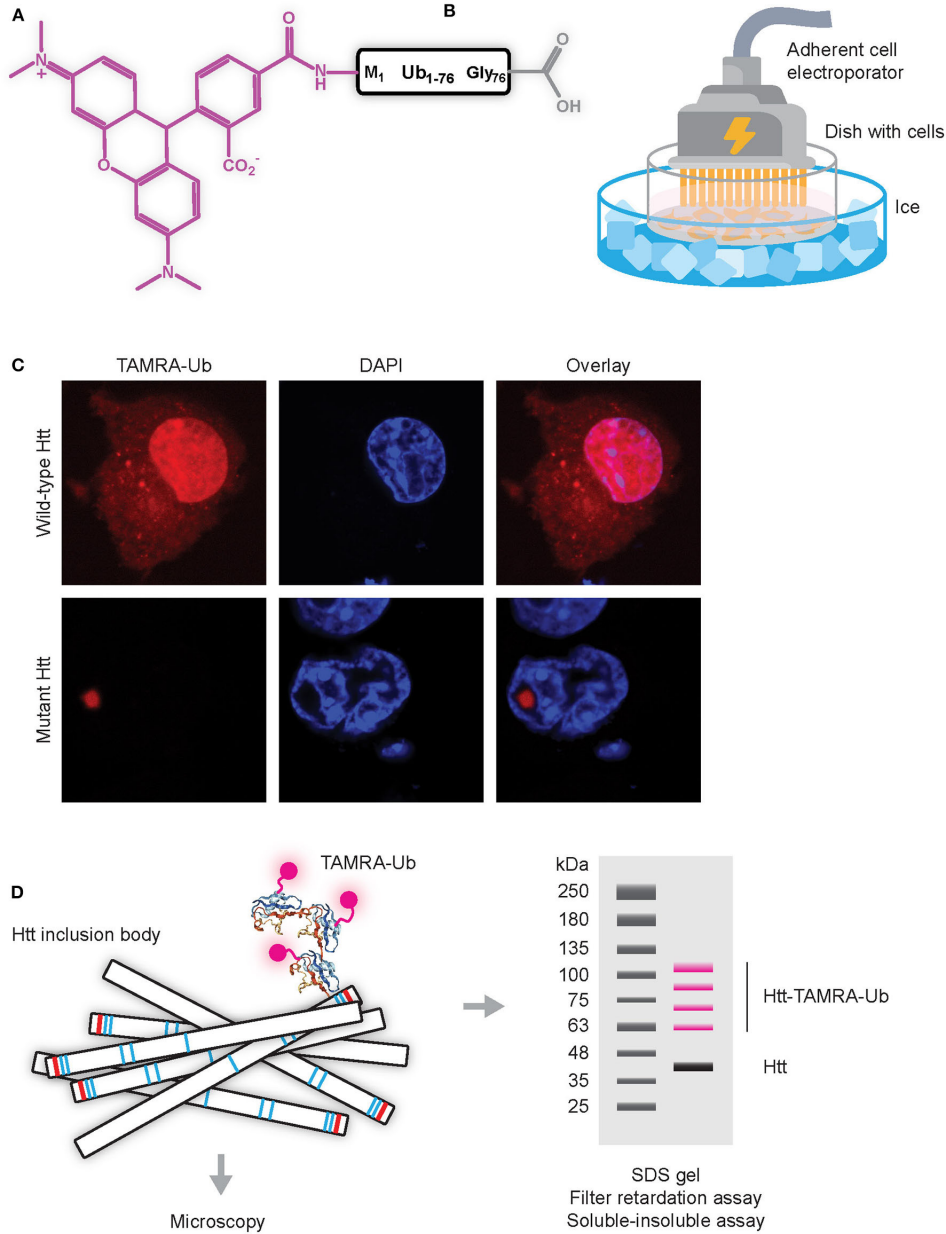
Application of TAMRA-Ub to study ubiquitination of mHtt aggregates **(A)** Scheme of TAMRA-Ub with Ub being fluorescently labeled on the N-terminus with TAMRA (5-tetramethylrhodamine, excitation 550 nm, emission 590 nm). **(B)** Electroporation of adherent cells with TAMRA-Ub dissolved in mannitol buffer, placed on ice to prevent intravesicular staining due to uptake by endocytosis. **(C)** TAMRA-Ub electroporated into cells is mainly localized in the nucleus and present on vesicles, while upon mHtt expression most TAMRA-Ub is recruited into the mHtt aggregate. **(D)** Upon electroporation of TAMRA-Ub, cells can be lysed and aggregates can be solubilized and separated by SDS-PAGE, with fluorescent bands representing TAMRA-Ub conjugated proteins.

Initial experiments to study dynamics of Ub recruitment to mHtt IBs were done using GFP or YFP-tagged ubiquitin combined with Fluorescent Recovery After Photobleaching (FRAP) protocols in living cells. Here, a small region of the fluorescent aggregate is photobleached and recovery of fluorescence is monitored in time. Since photobleaching is permanent, recovery can only occur when bleached proteins exchange with fluorescent proteins from the surroundings by diffusion or active transport. Since no recovery was observed when fluorescently-tagged Ub present in IBs was photobleached, these studies indicate that Ub is irreversibly sequestered into IBs (Raspe et al., 2009; Bersuker et al., 2016). However, the commonly-used GFP (or variant) tags are relatively large as compared to the size of the labeled target protein, especially when considering that GFP is ~27 kDa in size, while ubiquitin is ~8.5 kDa. By using synthetic ubiquitin labeled at the N-terminus with Tetramethylrhodamine (TAMRA-Ub) and electroporated into living cells (Figures 3A,B) it was more recently shown that ubiquitin is reversibly recruited to mHtt IBs (Juenemann et al., 2018). The TAMRA label is with its mass of only ~0.5 kDa much smaller than GFP (and variants) tags and it was found that TAMRA-labeled Ub behaves more similar to endogenous Ub when compared to GFP-tagged Ub. While GFP-tagged Ub can be expressed following transfection of cells with cDNAs, the TAMRA-Ub has to enter the cell via micro-injection or electroporation. Electroporation involves the application of a very short electrical pulse (few microseconds or milliseconds) which disturbs the phospholipid bilayer and forms temporary small pores through which the TAMRA-Ub can enter the cell. Electroporation can be performed using a cuvette with cells in suspension, or alternatively an adherent cell electrode can be used to electroporate adherent cells on coverslips, allowing for immediate visualization by microscopy. Note that in both cases electroporation has to be performed at low temperature to prevent uptake of TAMRA-Ub via endocytosis, as resulting fluorescent puncta represent internalized TAMRA-Ub in vesicles instead of cytoplasmatic TAMRA-Ub being involved in mono-ubiquitination and internalization of endosomes. Upon electroporation, TAMRA-Ub is mainly present in the nucleus and on cytoplasmic vesicles, but when mHtt induced IBs are present most TAMRA-Ub is recruited to IBs (Figure 3C). Both TAMRA-Ub and endogenous Ub are present in the entire mHtt aggregates, including the inner core, and TAMRA-Ub was incorporated into Ub linkages including poly-ubiquitinated mHtt itself (Juenemann et al., 2018). This can be visualized by SDS-PAGE WB after lysing cells and solubilizing the insoluble fraction including aggregated mHtt (Figure 3D). FRAP experiments showed that TAMRA-Ub was reversibly recruited to mHtt IBs, which was prevented when either E1 ligase or DUB inhibitors were used (Juenemann et al., 2018). This indicates that (de)ubiquitinating enzymes recruited to IBs are involved in mHtt ubiquitination as well as of other proteins sequestered into IBs, resulting in ongoing ubiquitination and deubiquitination of proteins present in IBs with reversible recruitment of ubiquitin.

### Recruitment of Ubiquitin Related Enzymes to Htt IBs

The reversible recruitment of Ub into mHtt IBs and the dependency on active E3 ligase and DUB activities as described above indicate that different E3 ligases and DUBs are recruited to aggregates. Indeed, several ubiquitin ligases and DUBs have been observed to co-localize with mHtt IBs, but only a few E3 ligases have so far been identified to affect mHtt aggregation, including Ube3a, CHIP, WWP1, and UBR5. Ube3A is an E3 ligase known to promote proteasomal degradation of misfolded proteins by enhancing K48-linkage type poly-ubiquitination, and Ube3A protein levels were found to be reduced during aging. When Ube3A levels were selectively decreased in HD mouse brains an increase in aggregate formation was observed combined with reduced ubiquitination of the IBs (Maheshwari et al., 2014). In contrast, overexpression of Ube3A reduced mHtt accumulation and aggregation (Bhat et al., 2014), suggesting that Ube3A is at least involved in ubiquitination of mHtt or sequestered proteins. Another E3 ligase that is recruited to mHtt IBs is the C-terminus of Hsc70-interacting protein (CHIP). Both the ubiquitination and the rate of degradation of mHtt was increased when CHIP was transiently overexpressed in cells, while aggregation and cell death were reduced (Jana et al., 2005). *In vivo* studies using mice that were haploinsufficient for CHIP showed an accelerated HD disease phenotype, while overexpression of CHIP showed a reduction in mHtt aggregates in zebra fish models of HD (Miller et al., 2005). Similar to Ube3A and CHIP, also the E3 ligase WPP1 is recruited to mHtt aggregates, but its activity appears to enhance mHtt levels and aggregation in both *in vivo* and *in vitro* models. This may be the result of ineffective ubiquitination as WWP1 ubiquitinates mHtt at an atypical position of Lys-63, which may impair efficient targeting to the UPS (Lin et al., 2016). More recently the role of UBR5 in mHtt IB formation was demonstrated, which appears to be required for proteasomal degradation of both normal and mutant Htt. Knockdown of UBR5 increased mHtt aggregation and neurotoxicity in invertebrate models, while loss of UBR5 increased Htt levels and IB formation in iPSCs expressing mHtt (Koyuncu et al., 2018). Intriguingly, this effect may be mediated by UBR5-mediated heterotypic ubiquitin K11/K48-linked chains that are also present in mHtt IBs, and which promote rapid proteasomal clearance of aggregation-prone proteins (Yau et al., 2017). Besides these E3 ligases also the E2 enzyme Ube2W has been reported to affect mHtt aggregation (Wang et al., 2018), which could be the result of the preference of Ube2W to ubiquitinate proteins with a disordered N-terminus.

The recruitment of E3 ligases and DUBs to mHtt IBs is likely to be due to the presence of numerous target proteins present in IBs either due to active recruitment or sequestration. By using fluorescent reporter proteins that can become reversibly and conditionally misfolded it was shown that the misfolded conformation of the reporter was targeted for ubiquitin-dependent degradation unless mHtt aggregates were present, resulting in the sequestration of these reporter proteins (Bersuker et al., 2016). Interestingly, only reporter constructs in a misfolded conformation accumulated at mHtt IBs, whereas constitutively ubiquitinated reporters which were not misfolded were not recruited to mHtt IBs. These data suggest that the folding state of a protein is the primary determinant for sequestration of proteins in an IB, instead of active recruitment of ubiquitinated proteins toward mHtt IBs. This reporter system nicely shows that the sequestration of misfolded proteins, possibly in a promiscuous fashion, contributes to mHtt aggregation. However, several recruited (de)ubiquitinating enzymes appear to be functional and enzymatically active as shown by using activity-based probes (ABPs) to label recruited enzymes. ABPs target only the active form of an enzyme, which allows for the identification and quantification of the pool of active enzymes in a biological sample. The development and application of ABPs for protein (de)ubiquitination was recently reviewed by Mulder and colleagues (Mulder et al., 2019). Recent examples for the usage of two chemically synthesized ABPs in HD are Cy5-Ub-Dha and Cy5-Ub-PA. Cy5-Ub-Dha is an ABP which reacts with active cysteine residues of E1, E2, and E3 enzymes and can irreversibly label active enzymes involved in protein ubiquitination (Mulder et al., 2016). Cy5-Ub-PA is a specific inhibitor of DUBs of the UCH, USP, and OTU DUB families, and fluorescently labels them (Ekkebus et al., 2013). Similar to TAMRA-Ub, most ABPs are not cell permeable and electroporation was used to introduce the ABPs into cells. These probes were electroporated into cells expressing GFP-tagged mHtt-exon1 to visualize aggregates. While the Cy5-Ub-Dha staining for active E1, E2, and E3 enzymes showed staining of entire aggregates, the Cy5-Ub-PA staining was present as a ring around the aggregates (Juenemann et al., 2018), suggesting that DUBs were mainly active in the periphery of mHtt IBs, while ubiquitinating enzymes were also active in the core of Htt IBs. Similarly, when using GFP-tagged enzymes, GFP-tagged ubiquitin ligase NEDD4.1 showed a complete co-localization with Htt aggregate staining, while co-transfection of GFP-tagged DUB USP5 resulted in a ring-formed staining around the aggregates (Juenemann et al., 2018). Together these data suggest that enzymes are active at mHtt aggregates, and that active ubiquitin ligases are found in both the core and periphery of IBs, while DUBs were found to be active at the periphery.

### Detecting Polyubiquitination of the Htt Protein

#### Degradation-Associated Polyubiquitination in HD Pathology

Mass spectrometry is an important tool to characterize ubiquitin association sites, and to distinguish different forms of ubiquitination, such as mono- and polyubiquitination. Whereas, tryptic peptides derived from mono-ubiquitin contain only unbranched peptides, tryptic peptides derived from polyubiquitin chains contain branched peptides as a result of isopeptide-bond formation between the C-terminal glycine residue of one ubiquitin molecule and the internal lysine residue of the other ubiquitin molecule. The ubiquitin-AQUA method was developed for the absolute quantification of ubiquitin, using isotope-labeled internal standard peptides for all seven possible polyubiquitin chain linkages, as well as internal standard peptides specific for mono-ubiquitination (Kirkpatrick et al., 2006). When known amounts of (heavy) isotope-labeled internal standard peptides were added to (light) samples, heavy and light peptides were subsequently separated by reversed phase high-performance liquid chromatography (HPLC) and analyzed by a targeted quantitative mass spectrometry approach named multiplexed Selected Reaction Monitoring (SRM). With this method one can measure the amount of total ubiquitin, the amount of monoubiquitin and polyubiquitin, as well as the amount of targeted substrate. This technique has been applied to study the function of the UPS in HD, using the UBA domain from human ubiquilin 2 (UBQLN2) to capture polyubiquitin chains from HD samples (Bennett et al., 2007). Here, polypeptides were eluted, digested with trypsin and measured by mass spectrometry and the abundance of the ubiquitin K48 diGly peptide was compared with a heavy labeled spiked-in peptide. K48 diGly peptide abundance was used as a measure of the UPS functioning as an increase in abundance of the ubiquitin K48 diGly peptide correlated with an increase in MG132 proteasome inhibitor concentration in cell culture. The authors observed an increase in the pool of K48 polyubiquitin linkages during HD pathology while the increase in the unconjugated ubiquitin pool was minimal or absent. In R6/2 HD mice expressing expanded mHtt exon1, elevated levels of K48 polyubiquitin linkages were first detected in cortex and striatum of 6 weeks old mice. Furthermore, a small increase in K48 polyubiquitin linkages was observed in 22 months old *Hdh*^*Q*150/*Q*150^ “knock-in” HD mice expressing full-length mHtt. Finally, half of the tested human HD cortex and striatum samples showed increased levels of the UbK48 isopeptide as compared to control (Bennett et al., 2007). Together these results suggest that the proteasome function is impaired during HD and that levels of polyubiquitin K48 linkages could be used as a biomarker for UPS impairment.

A recent global proteome and global ubiquitinome analysis of brain tissues of 40 weeks old homozygous Q175FDN mice that express full-length Htt with 175 polyQ repeats, and Q20 wild-type mice as control, used the diGly remnant of ubiquitin left on protein substrates after trypsin digestion to enrich peptides derived from ubiquitinated proteins using a specific antibody. When label-free quantification was used to compare relative protein and diGly peptide levels between HD and wild-type samples, increased levels of the ubiquitin protein and of ubiquitin K48-diGly peptides were observed in the insoluble fraction of HD mice brains (Sap et al., 2019). A similar result was obtained when HA-tagged ubiquitin mutants were transfected into cells expressing mHtt. These ubiquitin mutants contained lysine-to-arginine substitutions at all lysine residues except for either K48 or K63 and can therefore only make K48- or K63-linked polyubiquitin chains. Upon co-transfection of these mutants with mHtt into cells only co-localization of Htt-positive inclusions and K48 polyubiquitin was observed by fluorescence microscopy following immunostaining for K48 and K63 polyubiquitin, indicating that mutant Htt inclusions are K48-polyubiquitinated (Kah et al., 2005). Interestingly, mHtt IBs were also shown to be ubiquitinated via K11/K48 branched chains, which are mainly associated with enhanced degradation of regulators of mitosis by the proteasome. Heterotypic ubiquitination has been difficult to study, and to identify these branched chains bispecific antibodies were developed that detect K11 and K48 polyubiquitin linkages which are in close proximity, which allows for the identification of K11/K48 heterotypic polyubiquitin chains (Yau et al., 2017). Heterotypic chains adopt a branched or mixed topology due to ubiquitin-modification of more than one site on a single ubiquitin molecule. K11/K48-branched chains were identified on mitotic regulators, misfolded nascent proteins but also on mutant Htt, as fluorescent staining with the bispecific K11/K48 antibody co-localized with Htt-Q73-GFP aggregate staining in HeLa cells, embryonic stem cells and in differentiated neurons as observed by microscopy. Also aggregates of Q175 mHtt in mice brain were recognized by the K11/K48 bispecific antibody. Furthermore, purification of His-tagged wtHtt and mHtt from HeLa cell lysates under denaturing conditions followed by SDS-PAGE WB revealed that primarily mHtt samples were polyubiquitinated with K11- and K48-polyubiquitin linkages. The branched chains on mHtt were confirmed by using an ubiquitin^TEV/FLAG^ system, which results in ~2 kDa stamps on wild-type ubiquitin molecules whereby the presence of two or more stamps on one ubiquitin molecule indicates branched chains (Meyer and Rape, 2014; Yau et al., 2017). Also, proteins with a role in the K11/K48 quality control pathway co-localized with Q73 mHtt aggregates in HeLa cells, such as BAG6, p97/VCP, UBQLN2, and P62/SQSTM1, as was shown by microscopy. Inhibition of protein degradation and inhibition of the stabilization of newly synthesized misfolded proteins led to a loss of co-localization of K11/K48 Ub with Htt aggregates. In contrast, co-localization was maintained when new protein synthesis was blocked during these treatments. These results suggest that mHtt aggregates compete with newly synthesized misfolded proteins for a limited pool of enzymes involved in the Ub K11/K48-specific quality control system, including ubiquitin E3 ligases UBR4 and UBR5, ubiquitin-selective segregase P97/VCP and proteasome shuttles of the UBQLN family.

#### Atypical Forms of Polyubiquitination in HD

Also other polyubiquitin linkages have been associated with mHtt IBs. E3 ubiquitin ligase TRAF6 was found to be localized with mHtt IBs, especially at the outside. Overexpression of ubiquitin mutants which could only make homotypic chains revealed that TRAF6 facilitates atypical polyubiquitination of wildtype and mutant Htt with K6, K27, or K29 polyubiquitin chains. TRAF6 expression also enhanced aggregate formation and atypical ubiquitination of mHtt aggregates (Zucchelli et al., 2011). Htt aggregates were shown to be modified with linear M1-linked polyubiquitin chains (van Well et al., 2019). Usually ubiquitin associates with its C-terminal glycine residue to a lysine residue on a target protein, but in M1-linked ubiquitination the protein's N-terminal methionine is the target for ubiquitination. A M1-linked polyubiquitin chain is then formed by incoming ubiquitin molecules that associate with their C-termini to the N-terminal methionine of the preceding ubiquitin molecule. N-terminal polyubiquitination is catalyzed by the linear ubiquitin chain assembly complex (LUBAC), which comprises HOIL-1, HOIL-1 interacting protein (HOIP) and SHARPIN (Kirisako et al., 2006). M1 polyubiquitination can be reversed by the DUBs CYLD and OTULIN (Komander et al., 2009; Keusekotten et al., 2013). Van Well and colleagues also observed recruitment of components of the LUBAC complex (responsible for linear ubiquitination) including HOIP, HOIL-1L, and Sharpin to mHtt aggregates, as well as the recruitment of proteins that are known to associate specifically to M1-linked ubiquitination such as NEMO (NF-κB essential modifier) and Optineurin. Several sample types were studied including human neuronal SH-SY5Y cells expressing wild-type or mHtt, human frontal cortex HD samples and cortex and striatum of R6/2 HD mice. Both soluble and insoluble mHtt appeared to be directly ubiquitinated with linear ubiquitin as shown by immunoprecipitation of HA-tagged Htt exon1 and 3xKR variants of Htt-exon1. In addition, sequestration of transcription factor Sp1 to mHtt aggregates was affected by the levels of linear ubiquitin. Results of this study suggest that linear ubiquitination prevents unfavorable protein sequestration in aggregates, and promotes the degradation of misfolded proteins in a P97/VCP- and proteasome-dependent manner.

## Future Perspectives

While many aspects of the role of ubiquitination in HD, including identification of Htt ubiquitination sites, dynamics of ubiquitin recruitment to IBs, and alterations in the ubiquitinome during HD progression can be studied with current methodologies, various remaining challenges remain to be addressed. These include the quantification of low levels of polyubiquitination and the identification of polyubiquitin linkages as well as the identification of involved (de)ubiquitinating enzymes.

### Identification of Ubiquitin Ligases Involved in Htt Ubiquitination

Standard techniques for the identification of ubiquitin ligases specific for Htt would include microscopy-based co-localization assays, siRNA knockdown screens to determine the effect of knockdown of individual E3 ligases on soluble and insoluble mHtt levels, or the identification of a physical interaction using a yeast two hybrid assay or affinity-purification followed by mass spectrometry. Immunoprecipitation assays rely on the specificity of the antibody and the stable interaction between proteins throughout the procedure. There are a number of challenges when aiming to identify a specific E3 ligase. First of all is the typically very transient nature of their interaction (Pierce et al., 2009), which makes it difficult to identify these interactions with the standard techniques. Secondly, the rapid targeting of ubiquitinated substrates for protein degradation decreases the abundance of ligase substrates in the sample. Thirdly, ubiquitination is a reversible modification and rapid deubiquitination can make it challenging to identify ubiquitinated substrates. Finally, substrates could be ubiquitinated by different ubiquitin ligases, which hampers identification of involved E3 ligases by selective knockdown. Several novel approaches may enhance the possibility to identify new ubiquitin ligase-substrate pairs, including BioID and APEX.

#### Proximity-Dependent Biotin Identification (BioID)

**Figure 4:**
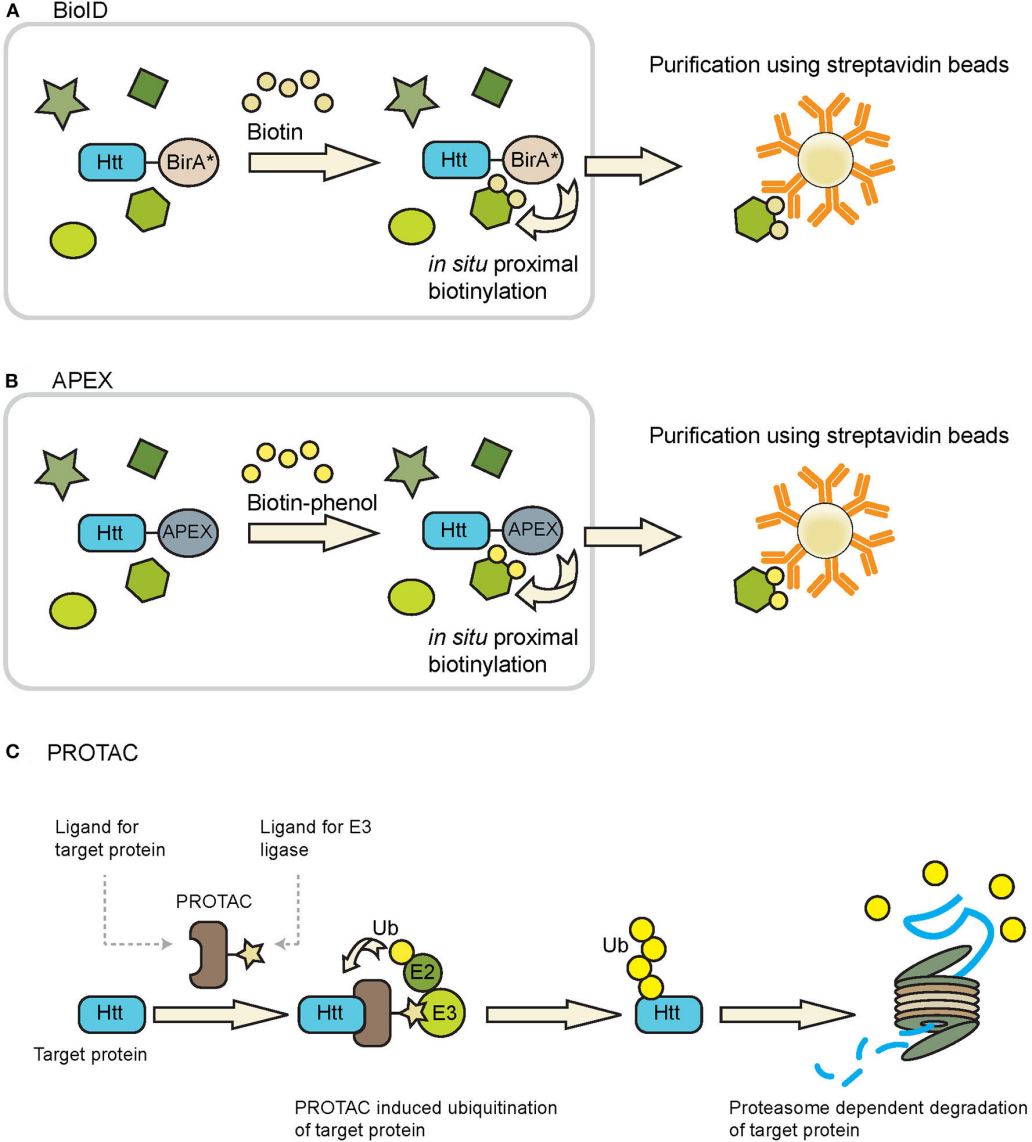
Tools that can be used to study Htt ubiquitination and degradation. **(A)** BioID and **(B)** APEX can be used to identify ubiquitin ligases and DUBs that putatively interact with Htt, as both techniques identify transient interacting proteins via promiscuous biotinylation of proteins in close proximity of the BirA* or APEX fusion protein. Biotinylated proteins can be purified using avidin/streptavidin beads and are subsequently identified by mass spectrometry. Figures adapted from Ueda et al. (2015) and Chu et al. (2017). **(C)** PROTACs can be used to induce ubiquitination of a protein of interest followed by proteasome-dependent degradation. PROTACs bring together the protein of interest (Htt in this case) and a ubiquitin ligase, leading to ubiquitination of the protein of interest and subsequent proteasome-dependent degradation.

BioID can be used to identify proteins in close proximity of a protein of interest in living cells (Roux et al., 2012, 2018). This approach makes use of the fusion of a protein of interest with a mutant form of the biotin ligase enzyme BirA (BirA^*^), which has been engineered in such a way that it can biotinylate neighboring proteins in a promiscuous fashion. Upon addition of biotin, proteins which are in close proximity (~10 nm) of the fusion protein are biotinylated and these could be efficiently enriched from samples due to the strong interaction of biotin with avidin/streptavidin conjugated beads (Figure 4A), which also makes it possible to study insoluble proteins, while a spatial resolution can be obtained by targeting BirA^*^ to a location-specific protein. Additionally, with the use of split-BioID constructs, consisting of a split BirA^*^ molecule, it is possible to confirm the interaction of two proteins of interest while also biotinylating other members of the protein complex (Schopp et al., 2017). After enrichment of the biotinylated proteins mass spectrometry is used to identify the interactomes of the fusion proteins. The advantages of BioID are the identification of weak and/or transient protein-protein interactions of both soluble and insoluble proteins, and the possibility to study protein-protein interactions at specific subcellular localizations. Disadvantages of the BioID approach include fusion of BirA^*^ to the protein of interest which accounts for an additional mass of ~35 kDa, which could affect the structure, function and localization of the protein of interest. However, a smaller and improved version of BioID, named BioID2, is also available (Kim et al., 2016). Whether the optimal location of the BirA^*^ is at the N-terminal or C-terminal side of the protein of interest will depend on the target protein. The expression of the fusion protein should ideally be done at endogenous levels, as high expression levels, as for instance obtained via transient expression systems, might result in increased levels of false positives. Hence expression under the control of an endogenous promotor is advised. Another limitation of this method is the relatively slow labeling kinetics of the BioID, which results in labeling timeframes of typically 12–24 h (Varnaite and MacNeill, 2016). However, also improved versions, called TurboID and miniTurbo, were recently developed and can label neighboring proteins within 10 min (Branon et al., 2018). BioID experiments could result in a large number of unspecific hits, which makes selection of the right control experiments important in order to discriminate between specific and unspecific interactors. Negative controls that could be used are either the parental cell line, or the expression of an epitope-tagged BirA^*^ protein at similar levels as the BirA^*^ fusion protein (Lambert et al., 2015). Quantitative proteomics could be used in combination with BioID in order to eliminate background proteins. BioID has been successfully applied to identify substrates of E2 ubiquitin conjugating and E3 ubiquitin ligase enzymes (Coyaud et al., 2015; Bakos et al., 2018; Dho et al., 2019). The fusion of a BioID molecule with wtHtt or mHtt might lead to the identification of novel enzymes with a role in the ubiquitin pathway.

#### Engineered Ascorbate Peroxidase (APEX)

A functionally related method to BioID is the 28 kDa APEX (Rhee et al., 2013). Upon treatment with hydrogen peroxide and biotin-phenol, APEX catalyzes the oxidation of biotin-phenol to generate the short-lived biotin-phenoxyl radical, which results in the biotinylation of proteins in close proximity to APEX. An improved version of APEX, APEX2, exhibits a higher sensitivity as compared to the original (Lam et al., 2015). Similar as with the BioID approach the biotinylated proteins can be enriched with streptavidin beads and subsequently identified by mass spectrometry (Figure 4B). APEX labeling times as short as 1 min could be used to biotinylate neighboring proteins, making it a much faster method as compared to BioID. This higher temporal resolution makes it possible to study interactome dynamics over time. Disadvantages of APEX are the fusion of a tag with the protein of interest, as well as the toxic effect that the biotin-phenol reagent can have in living cells, making it unsuitable for *in vivo* studies (Che and Khavari, 2017).

### Capturing Polyubiquitinated Proteins by Tandem Ubiquitin Binding Entities (TUBEs)

TUBEs can be used to enrich polyubiquitinated substrates from the cell (Hjerpe et al., 2009). TUBEs consist of four UBA domains separated by flexible linkers and fused to a tag or agarose bead to facilitate purification and detection. They have a higher affinity for polyubiquitin as compared to single UBA domains, and can be expressed in cell lines where they associate with polyubiquitinated proteins and protect them from proteasomal degradation as well as from the action of DUBs, thus stabilizing polyubiquitinated proteins. These polyubiquitinated proteins can subsequently be enriched by standard immunoprecipitations. Currently, TUBEs with specificity for M1, K48, and K63 homotypic polyubiquitin chains are available. A disadvantage of TUBEs is that they recognize homotypic polyubiquitin chains and it is not clear which part of the ubiquitin landscape consists of homotypic chains vs. mixed and branched polyubiquitin chains. Trypsin-resistant TUBEs (TR-TUBEs) could be used for protein identification by mass spectrometry, as otherwise high abundant peptides derived from the TUBE proteins will not be generated and measured by mass spectrometry. The application of TUBEs might increase the pool ubiquitinated Htt species by protecting them from the action of DUBs and proteasomal degradation, which might facilitate the identification of novel Htt ubiquitination sites or might shed more light on the regulation of K48-linked polyubiquitin targeting for degradation by the UPS.

### Targeted Protein Degradation by Proteolysis Targeting Chimeras (PROTACs)

Therapeutic strategies for HD focus on mHtt lowering in order to slow down or delay the onset of disease, either by reducing synthesis of mHtt or by accelerating the turnover of mHtt prior to aggregation. Various antisense oligonucleotide approaches are entering the clinic with the aim to reduce Htt mRNA levels, often requiring invasive approaches including repeated CSF injections or direct delivery of AAV viruses in the brain. Alternatively, one could aim to reduce mHtt protein levels by improving the turnover of mHtt via the UPS. This would require the identification of involved ubiquitinating enzymes and manipulating their activities.

Novel strategies also include the development of PROTACs to induce proteasome-dependent protein degradation via a targeting molecule (reviewed by Gu et al., 2018; Schapira et al., 2019; Zou et al., 2019). The designed hybrid molecule contains a moiety to recognize the substrate protein, and another moiety to recruit an E3 ligase to induce ubiquitination and subsequent proteasome-dependent degradation (Figure 4C). In the case of HD, one could use moieties recognizing the expanded polyQ tract of mHtt, or other Htt-specific domains that are accessible for PROTAC molecules. The challenge will be to generate a ligand with sufficient specificity for mHtt over wtHtt or other proteins with a polyQ repeat. Both peptide-based and small molecule-based PROTACs have been developed to recruit E3 ligases, with the latter being preferred as they can easier enter the human body (An and Fu, 2018). An important benefit of the PROTAC approach is that it is not required to identify the natural E3 ligase that targets Htt, as recruiting an E3 ligase to a substrate is often sufficient to trigger activity. In the case of HD, an E3 ligase able to generate K48-linkages might be sufficient, or E3 ligases like UBR5 to generate bi-specific K11/K48 linkages for accelerated degradation. In this way, the PROTAC takes advantage of the intracellular proteostasis pathways to selectively target and degrade disease-related proteins. Yet while representing a short-cut to accelerate Htt turnover via ubiquitination, PROTAC strategies may also encounter problems with permeability (especially by crossing the blood-brain barrier), off-target effects and stability.

### Final Remarks

The toolbox to study ubiquitination in HD is quickly expanding with new technologies including the possibility to use fluorescent activity probes to detect alterations in (de)ubiquitinating enzyme activities, new developments in proteomics to study alterations in the ubiquitinome, and chemical synthesis of ubiquitin with a variety of modifications including fluorophores and linkages. The possibility to synthesize Htt peptides with PTMs and the advances made in the field of chemical derived ubiquitin tools opens the door to generate specialized Ub-Htt peptides able to trap and identify enzymes involved in Htt ubiquitination. Together this will allow examination of the complexity of PTM crosstalk in HD and the intriguing role of the UPS in HD in more depth, which may lead to novel therapeutic strategies to lower mHtt levels and delay onset and progression of disease.

## Author Contributions

KS and ER wrote the manuscript and generated the figures.

## Conflict of Interest

The authors declare that the research was conducted in the absence of any commercial or financial relationships that could be construed as a potential conflict of interest.

## Abbreviations

ABPs: activity-based probes
APEX: engineered ascorbate peroxidase
BioID: proximity-dependent biotin identification
DUBs: deubiquitinating enzymes
HD: Huntington's disease
Htt: huntingtin
IB: inclusion body
iPSCs: induced pluripotent stem cells
mHtt: mutant huntingtin
PolyQ: polyglutamine
PROTACs: proteolysis targeting chimeras
PTM: post-translational modification
UBA: ubiquitin-associated domain
UPS: ubiquitin-proteasome system.
